# Supplementary material for: Accessibility measurement of highway transportation networks based on closeness-accessibility
Source: PLoS One. 2025 Nov 19;20(11):e0336928. doi: 10.1371/journal.pone.0336928 (PMC12629455; doi:10.1371/journal.pone.0336928)
Supplement: Attachment — Submitted filename: Response_to_Reviewers_auresp_2.doc [file pone.0336928.s003.doc]

**Response to the Reviewers’ Comments**

**Submission ID:** PONE-D-25-21841

**Title:** Accessibility measurement of highway transportation networks based on closeness-accessibility

We thank all the editors and reviewers for their valuable comments and suggestions. We have carefully revised the manuscript to enhance its clarity and facilitate the understanding of the readers. Our point-to-point responses are presented in the following. We hope that the revision would satisfactorily address the comments and concern of the editors and reviewers.

**Reviewer #1:**

We sincerely thank you for the valuable feedback that we have used to improve the quality of our manuscript.

**Reviewer #2:**

**It seems authors addressed previous comments. I would like to recommend to published articles after minor improvements, such as improving resolution of imagers.**

**Response：**We are very grateful to the reviewer for the positive evaluation and the recommendation for publication. We also appreciate the constructive feedback on the image quality. Following the reviewer’s suggestion, we have carefully improved the resolution of all figures in the manuscript to ensure they are clear and meet the publication standards. All figures have been regenerated and exported at a high resolution (600 DPI) by the Preflight Analysis and Conversion Engine (PACE) digital diagnostic tool.

We have also taken this opportunity to proofread the entire manuscript again to correct any minor typographical errors. Thank you once again for your valuable time and guidance.

**Reviewer #3:**

We sincerely thank you for the valuable feedback that we have used to improve the quality of our manuscript.The comments are laid out below in **bold** font and specific concerns have been numbered. Our response is given in **purple** font and changes to the manuscript are highlighted in **yellow**.

**1. Still the section on the objectives of the research after introduction section clearly stating the need, Objectives, Scope and limitations of the study. In line 80-85 Page 17 in introduction section what you have written is Aim of research which is generalwhich objectives are specific.**

**Response：**Thanks for the comment. As you suggested, we have adjusted the introduction section of the article to clarify the aim of research in line 80-85 page 4 , and we also copy here:

*“……The study aims to quantify the impact of different travel demand patterns on the overall accessibility of the highway network. By comparing network performance in a real network, the importance of node centrality non-uniformity is evaluated. The proposed closeness-accessibility modeling bridge spatial interaction theory and network science. Our case study focuses on the three-level highway network of national, provincial and county highways in Liaoning province, utilizing only structural data and excluding any information about traffic flow.……”*
